# Multiple Doses of Erythropoietin Impair Liver Regeneration by Increasing TNF-α, the Bax to Bcl-xL Ratio and Apoptotic Cell Death

**DOI:** 10.1371/journal.pone.0003924

**Published:** 2008-12-11

**Authors:** Katja Klemm, Christian Eipel, Daniel Cantré, Kerstin Abshagen, Michael D. Menger, Brigitte Vollmar

**Affiliations:** 1 Institute for Experimental Surgery, University of Rostock, Rostock, Germany; 2 Institute for Clinical & Experimental Surgery, University of Saarland, Homburg, Germany; University Hospital of the Saarland, Germany

## Abstract

**Background:**

Liver resection and the use of small-for-size grafts are restricted by the necessity to provide a sufficient amount of functional liver mass. Only few promising strategies to maximize liver regeneration are available. Apart from its erythropoiesis-stimulating effect, erythropoietin (EPO) has meanwhile been recognized as mitogenic, tissue-protective, and anti-apoptotic pleiotropic cytokine. Thus, EPO may support regeneration of hepatic tissue.

**Methodology:**

Rats undergoing 68% hepatectomy received daily either high dose (5000 IU/kg bw iv) or low dose (500 IU/kg bw iv) recombinant human EPO or equal amounts of physiologic saline. Parameters of liver regeneration and hepatocellular apoptosis were assessed at 24 h, 48 h and 5 d after resection. In addition, red blood cell count, hematocrit and serum EPO levels as well as plasma concentrations of TNF-α and IL-6 were evaluated. Further, hepatic Bcl-x_L_ and Bax protein expression were analyzed by Western blot.

**Principal Findings:**

Administration of EPO significantly reduced the expression of PCNA at 24 h followed by a significant decrease in restitution of liver mass at day 5 after partial hepatectomy. EPO increased TNF-α levels and shifted the Bcl-x_L_ to Bax ratio towards the pro-apoptotic Bax resulting in significantly increased hepatocellular apoptosis.

**Conclusions:**

Multiple doses of EPO after partial hepatectomy increase hepatocellular apoptosis and impair liver regeneration in rats. Thus, careful consideration should be made in pre- and post-operative recombinant human EPO administration in the setting of liver resection and transplantation.

## Introduction

The liver is one of the most remarkable organs because of its endogenous property to proliferate and to fully regenerate. The process of liver regeneration after partial hepatectomy (pHx) includes the division of almost all hepatocytes with the goal of replacing the lost functional mass. Meanwhile, large amounts of information have become available about the underlying mechanisms of cell replication [1], [2]. It is common view that liver regeneration encompasses three pathways, i.e. cytokine, growth factor and metabolic, linking liver function with cell growth and proliferation [2]. The cytokines, growth factors and metabolic signals must delicately interact to coordinate gene expression during the immediate early response. A characteristic feature of the regenerative process is that all components of each pathway are required for optimal regeneration, though no single gene, factor or mediator can be considered mandatory and essential for liver regeneration [1].

Great advances in the understanding of the regeneration process encourage hepatologists and transplant surgeons to further propel living donor liver transplantation and extended liver resections. Thus, therapeutic strategies are needed, which allow for promoting growth of ‘small-for-size’ transplants or limited residual mass [3], [4]. In addition, diseased livers with compromised regeneration, such as cirrhotic or acute necrotic livers, would benefit from liver growth-enhancing strategies [5]. Within this context, recombinant human erythropoietin (rHuEPO) treatment seems to be a valid approach, because the drug is already available and has a distinct safety margin for human use [6]–[8]. Besides its hematopoietic function, EPO has meanwhile been recognized as an anti-apoptotic, mitogenic and tissue-protective pleiotropic cytokine [9]–[11]. Recent studies have identified multiple paracrine and autocrine functions of EPO and its analogues that coordinate local responses to injury in brain, kidney and myocardium by inhibition of apoptosis and augmentation of cellular regeneration [12]–[15].

Schmeding et al. demonstrated that portalvenous administration of EPO increased liver regeneration in rats after 70% liver resection [16]. However, the same group could not confirm their own previous observation of EPO on liver regeneration [17]. In the here presented study, we communicate that multiple doses of EPO is accompanied by release of TNF-α and delays restoration of liver mass. Underlying mechanisms are presented and will be discussed.

## Materials and Methods

### Animal model

Male Sprague-Dawley rats (body weight (bw) 250–350 g; Charles River Laboratories, Sulzfeld, Germany) were used. The experiments were conducted in accordance with the German legislation on protection of animals and the NIH Guide for the Care and Use of Laboratory Animals (Institute of Laboratory Animal Resources, National Research Council). Animals were housed in standard rooms of the central animal husbandry of our faculty with a 12 hour light-dark cycle and had free access to water and standard laboratory chow ad libitum.

For hepatectomy, animals were anesthetized by breathing isoflurane (1.6 vol%) in air and subjected to a 68% hepatectomy according to the method described by Higgins and Anderson [18]. In supine position, an upper midline incision of the abdomen was followed by retraction of the xyphoid cartilage for adequate exposure of the liver and division of hepatic ligaments. The right median, the left median and the left lateral lobes were ligated and removed, resulting in a 68% hepatectomy. After irrigation of the abdomen with warm saline, the peritoneum and the skin were closed with running 6–0 and 5-0 sutures, respectively. Postoperatively, animals were allowed to recover from anesthesia and had free access to food and water until the final experiment.

### Experimental groups and protocol

Animals received 5000 IU/kg bw EPO (high dose) intravenously every 24 h starting at −48 h prior to hepatectomy. Animals which daily received equivalent amounts of physiologic saline solution served as controls. At either 24 h, 48 h or 5 d after hepatectomy (n = 6–10 animals at each time point and group) animals were exsanguinated by cardiac puncture under pentobarbital anesthesia and blood as well as liver tissue was sampled for subsequent analysis. Wet weight of regenerated liver was used to calculate growth of residual liver lobes by the following formula: weight of regenerated liver/preoperative liver weight×100 (%). Preoperative liver weight was estimated as 3.75% of body weight [19].

In an additional set of experiments rats received EPO 500 IU/kg bw (low dose) intravenously every 24 h starting at −48 h prior to hepatectomy. At 24 h post hepatectomy, i.e. at maximum of DNA-synthesis [20], animals (n = 4) were killed and blood as well as liver tissue were sampled as described above. Hepatectomized animals which daily received equivalent amounts of physiologic saline solution served as controls (n = 4). To study liver cell proliferation 5-bromo-2′-deoxyuridine (BrdU, 50 mg/kg bw) was applied intraperitoneally 1 h before sacrifice.

For determination of physiological baseline values blood samples were obtained from non-resected, untreated rats (n = 5) via the tail vein under light isoflurane anesthesia.

### Laboratory analysis

Systemic blood cell counts were measured using a Coulter Counter (AcTdiff, Coulter, Hamburg, Germany). Moreover, blood samples served for analysis of EPO concentrations using chemiluminescence enzyme immuno assay (DPC, Bad Nauheim, Germany). EDTA plasma further served for spectrophotometrical determination of alanine aminotransferase (ALT) as well as for analysis of tumor necrosis factor (TNF)-α and interleukin (IL)-6, using commercially available enzyme-linked immunosorbent assay (ELISA) kits in accordance to the manufacturer's instructions (Pierce Biotechnology, Rockford, IL 61105, USA).

### Histology and immunohistochemistry of liver tissue

Remnant liver tissue was excised at 24 h, 48 h and 5 d after resection, fixed in 4% phosphate-buffered formalin for 2 to 3 days and then embedded in paraffin. For immunohistochemical demonstration of PCNA and BrdU, 4 µm sections collected on poly-L-lysine-coated glass slides were treated by microwave for antigen unmasking. Mouse monoclonal anti-PCNA (1:100; Novocastra Laboratories, Newcastle upon Tyne, UK) and mouse monoclonal anti-BrdU (1:50; Dako Cytomation, Hamburg, Germany) were used as primary antibodies and incubated for 18 h at 4°C. After equilibrating to room temperature, sections were incubated with horseradish peroxidase-conjugated goat anti-mouse IgG (1:100) for 30 min (Dako Cytomation). Fuchsin (PCNA) or 3,3′-diaminobenzidine (BrdU) were used as chromogens. The sections were counterstained with hemalaun and examined by light microscopy (Axioskop 40, Zeiss, Göttingen, Germany). PCNA- and BrdU-positive nuclei were counted within 40 consecutive high power fields (x40 objective, numerical aperture 0.65) and are given as positive cells per mm^2^. For quantification of apoptotic cell death, 4 µm sections were mounted on poly-L-lysine glass slides and exposed to an apoptosis-specific staining kit (indirect in situ DNA nick end labeling (TUNEL) assay; ApopTag, Chemicon International, Inc., Temecula, CA, USA) according to the manufacturer's instructions. TUNEL-positive hepatocytes were counted within 40 consecutive high power fields and given as positive cells per mm^2^.

### Western Blot analysis

For Western blot analysis of Bcl-x_L_ and Bax, liver tissue was homogenized in lysis buffer (1 M Tris pH 7.5, 5 M NaCl, 250 mM EDTA, 10% Triton-X 100, 4% NaN_3_, and 100 mM PMSF), incubated for 30 min on ice, and centrifuged for 15 min at 10,000×g. Prior to use, all buffers received a protease inhibitor cocktail (1:100 vol/vol; Sigma, St. Louis, MO, USA). Protein concentrations were determined using the BCA protein assay (Pierce, Rockford, IL, USA) with bovine serum albumin as standard. Equal amounts of whole protein extracts (20 µg) were separated discontinuously on sodium dodecyl sulphate polyacrylamide gels (12% SDS-PAGE) and transferred to a polyvinyldifluoride membrane (Immobilon-P transfer membrane; Millipore, Billerica, MA, USA). After blockade of non-specific binding sites, membranes were incubated for 2 h at room temperature with a mouse monoclonal anti-Bcl-x_L_ (1:1,000; BD PharMingen, Heidelberg, Germany) or a mouse monoclonal anti-Bax antibody (1:250; BD PharMingen), followed by a secondary peroxidase-linked anti-mouse antibody (BcL-x_L_ 1:10,000 and Bax 1:20,000; Sigma). Protein expression was visualized by means of luminol enhanced chemiluminescence (ECL plus, Amersham Pharmacia Biotech, Freiburg, Germany) and digitized with ChemiDoc™ XRS System (Quantity One, Bio-Rad Laboratories GmbH, Munich, Germany). Signals were densitometrically assessed (Quantity One) and normalized to the β-actin signals as loading controls (mouse monoclonal anti-β-actin antibody, 1:20,000; Sigma).

### Statistical Analysis

Data are expressed as means±SEM. The Kolmogorov-Smirnov test was applied for analyzing the normality of the distribution of the parameters. The unpaired Student́s t-test was used to compare mean values between groups at the respective time point. The parameters that did not fit into normal distribution were compared using the Mann-Whitney rank sum test. Differences were considered statistically significant when P<0.05 (SigmaStat, Jandel Corporation, San Rafael, CA, USA).

## Results

### Systemic effects upon EPO administration

Daily administration of high dose EPO (5000 IU/kg bw) caused a massive rise of circulating EPO concentrations with a maximum value of almost 2500 mU/mL at 24 h after resection, while EPO was barely detectable in controls (table 1). EPO stimulated hematopoiesis in a time-dependent fashion with an increased hematocrit of 59% at 5 d after hepatectomy and an increased red blood cell (RBC) count of 9.3×10^12^/L compared to normal values of 40–45% and 6.9–7.5×10^12^/L in saline-treated controls (table 1). In contrast, administration of low dose EPO (500 IU/kg bw) led to an only slight increase of the hematocrit (47%) and the RBC count was not different compared to that measured in control animals (table 1).

**Table 1:** Serum EPO concentration, hematocrit and red blood cell count

	baseline	5000 IU/kg bw iv						500 IU/kg bw iv	
		24 h		48 h		5 d		24 h	
		Con	EPO	Con	EPO	Con	EPO	Con	EPO
serum EPO [mU/mL]	14.9±7.7	1.0±0.0	2453±167*	1.5±0.2	841±254*	1.1±0.4	726±400*	14.7±7.6	147±76*
hematocrit [%]	40.5±2.5	44.9±2.4	52.6±0.6*	45.0±1.9	55.1±1.0*	40.1±1.2	58.6±2.4*	42.4±2.5	47.3±4.1
RBC count [10^12^/L]	6.7±0.4	7.5±0.3	8.2±0.1*	7.1±0.3	8.2±0.2*	6.9±0.1	9.3±0.5*	7.0±0.4	7.3±0.7

Serum EPO concentration, hematocrit and red blood cell (RBC) count in animals at 24 h, 48 h and 5 d after pHx and daily administration of EPO at a dose of either 5000 IU/kg bw or 500 IU/kg bw. Control animals received equivalent volumes of physiologic saline (Con). For determination of physiological baseline values blood samples were obtained from non-resected, untreated rats (baseline). Data are given as means±SEM; Mann-Whitney rank sum test, ^*^ P<0.05 vs Con at the respective time point and EPO dose.

### Effect of high dose EPO on hepatocellular proliferation

**Figure 1:**
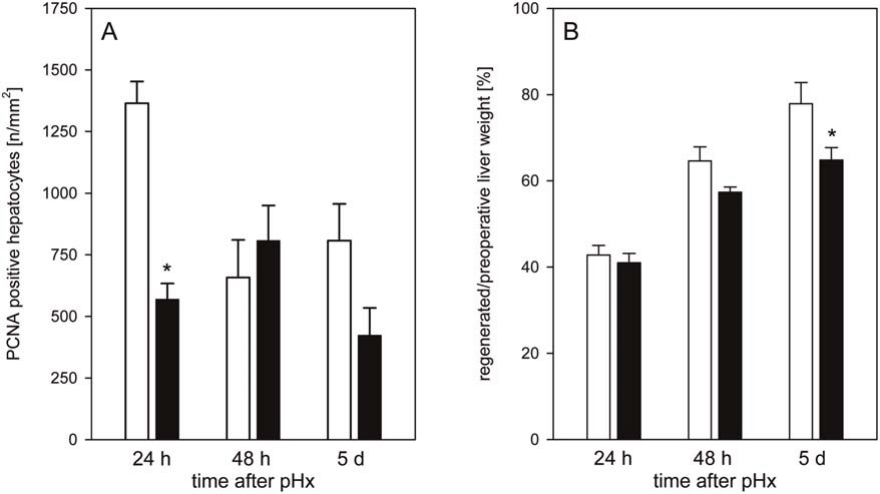
PCNA expressing hepatocytes and restoration of liver weight. Quantitative analysis of PCNA expressing hepatocytes (A) and restoration of liver weight (B) in animals at 24 h, 48 h and 5 d after pHx and daily administration of high dose EPO (5000 IU/kg bw iv; closed bars) or physiologic saline solution (open bars). PCNA expression was assessed by immunohistochemistry. The weight of the regenerating liver was used to calculate the growth of residual liver lobes. For detailed information please see section Material and Methods. Means±SEM; unpaired Student's t-test. * P<0.05 vs the saline-treated group at the respective time point.

To evaluate the influence of EPO on the regenerative capacity after pHx, livers were studied by immunohistochemistry for hepatocellular proliferation at 24 h, 48 h and 5 d after resection. PCNA labeling revealed a significant inhibition of hepatocellular proliferation in EPO-treated animals in particular during the initial 24-h-period after liver resection (figure 1A). As a consequence, restoration of liver weight after EPO administration was markedly delayed (figure 1B). Whereas control animals revealed a constant increase of liver mass upon pHx achieving ∼80% of preoperative liver weight at day 5 after resection, EPO-treated animals showed only a limited restoration up to ∼65% of the initial liver weight at day 5. This strongly underscores the deteriorating effect of high dose EPO on the process of liver regeneration.

### Effect of high dose EPO on hepatocellular apoptosis

**Figure 2:**
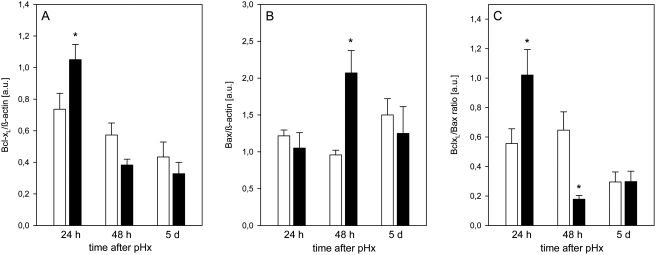
Anti-apoptotic Bcl-x_L_ and pro-apoptotic Bax as well as the Bcl-x_L_/Bax ratio. Protein expression of anti-apoptotic Bcl-x_L_ (A) and pro-apoptotic Bax (B) as well as the Bcl-x_L_/Bax ratio (C) in animals at 24 h, 48 h and 5 d after pHx and daily administration of high dose EPO (5000 IU/kg bw iv; closed bars) or physiologic saline solution (open bars). Data are normalized to ß-actin as loading control. Means±SEM; unpaired Student's t-test. * P<0.05 vs the saline-treated group at the respective time point.

**Figure 3:**
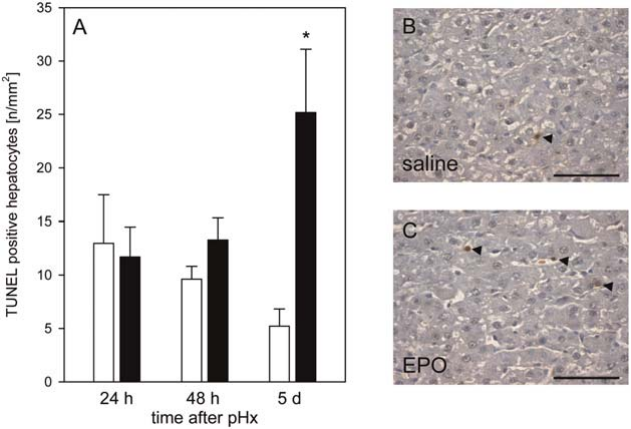
Hepatocellular apoptosis. Apoptotic cell death, as assessed by TUNEL-analysis in animals at 24 h, 48 h and 5 d after pHx and daily administration of high dose EPO (5000 IU/kg bw iv; closed bars) or physiologic saline solution (open bars) (A). Representative images at day 5 after pHx of a saline-treated control (B) and an EPO-treated animal (C). Arrowhead indicate TUNEL-positive apoptotic hepatocytes. Bars represent 50 µm. Means±SEM; unpaired Student's t-test. * P<0.05 vs the saline-treated group at the respective time point.

Western blot analysis showed that EPO administration led to a higher expression of the anti-apoptotic gene Bcl-x_L_ at 24 h after hepatectomy when compared with vehicle-treated controls (figure 2A). However, expression of Bcl-x_L_ decreased at the following days to equal levels in both groups (figure 2A). EPO-treated animals further showed a transient rise of the pro-apoptotic gene Bax at 48 h after hepatectomy, while expression of Bax remained unchanged in controls (figure 2B). Consequently, EPO-treated animals revealed a shift of the Bcl-x_L_/Bax ratio from a net anti-apoptotic at 24 h towards a net pro-apoptotic effect at 48 h (figure 2C). In line with this, control and EPO-treated animals demonstrated completely different patterns of hepatocellular apoptosis upon pHx (figure 3). Whereas control animals revealed decreasing numbers of TUNEL-positive hepatocytes from 13/mm^2^ at 24 h to 5/mm^2^ at 5 d after liver resection, high dose administration of EPO resulted in increasing numbers of apoptotic cells with ∼25/mm^2^ at day 5 (figure 3).

### Effect of high dose EPO on transaminase and cytokine release

**Figure 4:**
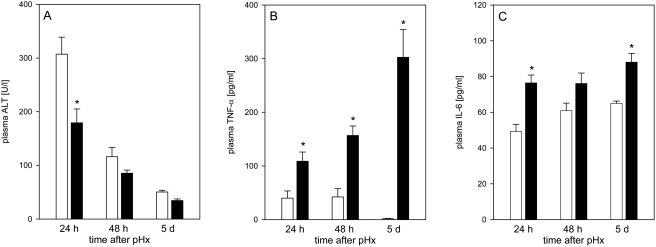
Transaminase and cytokine release. Plasma levels of ALT (A), TNF-α (B), and IL-6 (C) in animals at 24 h, 48 h and 5 d after pHx and daily administration of high dose EPO (5000 IU/kg bw iv; closed bars) or physiologic saline solution (open bars). Means±SEM; unpaired Student's t-test. * P<0.05 vs the saline-treated group at the respective time point.

Application of EPO significantly reduced systemic ALT release at 24 h after pHx compared to saline-treated controls (figure 4A). Partial hepatectomy was accompanied by a transient release of TNF-α of about 40 pg/mL at 24 h and 48 h after liver resection in control animals. At day 5 TNF-α was not anymore detectable. In contrast, EPO-treated animals revealed steadily increasing plasma levels of TNF-α from 100 pg/mL at 24 h to 300 pg/mL at day 5 after hepatectomy (figure 4B). Concomitantly, administration of EPO caused also an elevation of plasma IL-6 concentrations at all time points studied when compared with resected saline-treated animals (figure 4C).

### Effect of low dose EPO on hepatocellular proliferation

**Figure 5:**
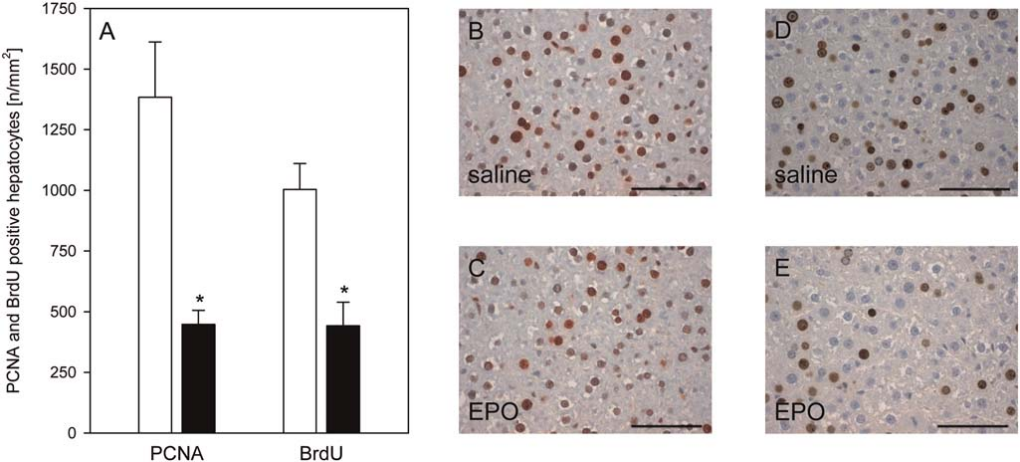
PCNA-expression and BrdU-incorporation. Quantitative analysis and representative images of PCNA expressing hepatocytes (A, B and C) and incorporation of BrdU (A, D and E) in animals at 24 h after pHx and daily administration of low dose of EPO (500 IU/kg bw iv; closed bars) or physiologic saline solution (open bars). Fuchsin-red (B and C) and DAB-brown (D and E) positive hepatocytes are PCNA-expressing and BrdU-incorporating cells, reflecting cells undergoing proliferation and DNA-synthesis. Note the marked reduction of these cells within the liver tissues of the EPO-treated animals (C and E). Bars represent 50 µm. Means±SEM; unpaired Student's t-test. * P<0.05 vs the saline-treated group.

In order to clarify, whether the impairment of liver regeneration after high dose EPO was induced by its pleiotropic, i.e. non-hematopoietic actions or just only by the increased hematocrit, an additional set of animals with administration of low dose EPO (500 IU/kg bw) was analyzed at the time point of maximal DNA synthesis, i.e. at 24 h after pHx. Analysis of both PCNA expression and BrdU incorporation revealed a statistically significant impairment of hepatocellular proliferation and DNA-synthesis in animals treated with the low dose of EPO when compared to saline-treated controls (figure 5).

## Discussion

The present study communicates the following major findings: (i) Daily administration of EPO in hepatectomized rats results in high circulating concentrations of EPO, which time-dependently induce erythropoiesis. (ii) EPO impairs liver cell proliferation upon pHx. (iii) EPO upregulates TNF-α and increases apoptotic cell death, most likely by shifting the Bcl-x_L_/Bax ratio towards a net pro-apoptotic effect.

Loss of liver cells caused by surgical resection, viral disease or chemical injury triggers replication of hepatocytes, which are capable of repopulating the liver. Nonetheless, conditions of compromised regeneration, such as acute liver failure, small-for-size transplants and liver cirrhosis, necessitate the development of therapeutic strategies to obtain rapid growth of the remnant liver. It has been shown that healthy live-liver donors did not achieve complete liver regeneration and ultimately reach only approximately 85% of the mass of the original liver during the first 12 months post-transplantation [3], [4], [21]. The incomplete or delayed regeneration of the liver bears the risk of having a reserve capacity deficit and thus might benefit from growth-supporting regimens. As production of EPO is generally suppressed following injury, administration of exogenous EPO seems to be a valid approach in this context. The observation that cyclosporine-based immunosuppression inhibits EPO production after liver transplantation [22] further warrants the application of EPO for hepatic growth support.

Herein, we show that administration of EPO delays liver regeneration after pHx. Daily administration of high dose EPO (5000 IU/kg bw) in hepatectomized rats reduces the expression of PCNA and results in a significantly diminished restoration of liver mass. Also, a ten-fold lower dose of EPO (500 IU/kg bw) led to decreased PCNA expression and a diminished DNA synthesis at the timepoint of maximal DNA-synthesis in comparison to animals without EPO treatment. Being aware that the dose of 5000 IU/kg bw is a very high one when compared with that used for treatment of anemia (10–15 IU/kg), one has to consider that similar doses have been used experimentally to exert cytoprotective effects in non-hematopoietic cells [23]. Dosing of 5000 IU/kg in rodents is comparable to the dose of about 500 IU/kg which has been tested in humans in a small clinical stroke [24], taking into account the different weight-to-surface ratio. In addition, recombinant human EPO displayed a 3-4 time extended half-life in humans compared to controls [25], [26]. The observed delay of hepatic regeneration is in contrast to a most recently published work by Schmeding et al. showing that administration of rHuEPO effectively increased liver regeneration in rats after 70% liver resection [16]. The discrepant results of the latter study and the present one may be related to differences in the route of EPO administration. Whereas in the present study EPO was applied daily via the tail vein, Schmeding and coworkers analyzed two different modalities namely subcutaneous and intraportalvenous EPO application, however, displaying identical effects for both study groups [16]. Unfortunately no data of EPO serum levels and hematocrit were presented. Schmeding et al. did not show whether the observation of enhanced regeneration capacity after 70% liver resection resulted in an increased restoration of liver mass. Moreover, in a most recently published work of the same group about a synergistic effect of EPO in combination with curcumin the authors could not confirm their previous result of EPO on liver regeneration [17]. These conflicting observations suggest caution in the use of EPO after liver resection.

Herein, we observed a cytoprotective effect of EPO during the first day after pHx, whereas EPO treatment resulted in a rise of apoptotic cell death during the late phase of the regeneration process. It has been shown, that EPO displays its protective effects in non-hematopoietic tissue by regulating the balance between pro- and anti-apoptotic Bcl-2 family proteins. Johnson et al. reported that in acute renal failure EPO decreased the expression of pro-apoptotic Bax and did not affect the expression of anti-apoptotic molecules Bcl-2 and Bcl-x_L_ [15]. On the other hand, EPO increased the expression of Bcl-xL in vitro [7] and the marked shift in Bcl to Bax ratio was consistent with an overall antiapoptotic effect of EPO on rat microglial cells [27]. Concomitantly, renoprotective [28] and neuroprotective [29] effects of EPO could be attributed to an upregulation of Bcl-2 and a downregulation of Bax. Though the relative balance of the Bax and Bcl-xL proteins seems to be a critical factor, the exact mechanism responsible for the anti-apoptotic efficacy of EPO is not yet clarified [30]. In the present study we could observe an initial upregulation of the anti-apoptotic member Bcl-x_L_ at 24 h after pHx in the EPO-treated group. However, the Bcl-x_L_ expression decreased to 50% at 48 h, whereas a concomitant increase of the pro-apoptotic Bax protein could be deteced. Along with this shift of the Bcl-x_L_/Bax ratio towards the pro-apoptotic Bax at 48 h, massive apoptosis occurred at day 5. To the best of our knowledge this is the first report of a proapoptotic effect after repetitive injection of EPO.

Astonishingly, EPO treatment in the setting of liver resection resulted in an increase of TNF-α, which is in contrast to previous studies showing that EPO exerts its tissue-protective effect in the liver through a reduction of the serum levels of several cytokines [31], [32]. It is to note that identical application of EPO in animals without liver resection did not result in a comparable elevation of TNF-α plasma levels (own unpublished observations). TNF-α exerts two paradoxical effects on the process of liver regeneration [33]. TNF-α released between 1 and 12 hours post hepatectomy initiates liver regeneration through its receptors located on hepatocytes and nonparenchymal cells. On the other hand, excessive TNF-α production between 12 and 24 hours after liver resection was considered to be a major determinant of liver failure [33]. TNF-α is mainly produced by Kupffer cells. As LPS is a potent stimulator of TNF-α release by Kupffer cells it has been speculated that resection-induced elevation of portal blood flow with increased load of LPS to the remnant liver provokes the Kupffer cells to release TNF-α [34]. However, the distinct stimulus for TNF-α release after liver resection is still unclear. Stimulation of p38 mitogen-activated protein (MAP) kinase has been reported to contribute to the stabilization of TNF-α mRNA in macrophages [35]. In addition, TNF-α augments itself in an autocrine fashion through activation of the p38 MAP kinase-related intracellular signal pathway [36]. EPO clearly activates the p38 signaling pathway [37] and exerts its protective effect amongst others via activation of p38 MAP kinase [38]. Thus, it might be supposed that both the initially elevated TNF-α plasma level after liver resection and the EPO application lead to an activation of p38 signaling pathway finally causing the unexpected surge of TNF-α.

The pro-apoptotic shift of the Bcl-x_L_/Bax ratio may result from increased levels of TNF-α. In support of this view, culture supernatants from activated Kupffer cells in a mouse tumor model were able to change the balance between Bax and Bcl-2 in favor of Bax [39]. Although TNF-α is thought to be an initial and mandatory cytokine for liver regeneration [40], Tsutsumi et al. showed that selective suppression of TNF-α and IL-1β could improve liver function and facilitate liver regeneration after extended hepatectomy [41]. Hepatocytes from TIMP3^−/−^ mice, which failed to control release of TNF-α, completed the cell cycle in a model of liver regeneration, but then underwent cell death owing to sustained activation of TNF-α [42]. Thus, it is reasonable to assume that the overwhelming release of TNF-α in the present study contributed to the impairment of liver regeneration.

Transgenic mice showing cerebal and systemic overexpression of EPO revealed an impaired outcome in a model of focal brain ischemia [43]. The authors have pointed out that chronic systemic treatment with EPO may deteriorate outcome after stroke either because of elevated hematocrit or other chronic effects [43]. We observed a significant increase of hematocrit and RBC count over the 5-day study period in the hepatectomized animals, which received the high dose of EPO. It is reasonable to speculate that this hematopoietic response with respect to pro-coagulant and pro-thrombotic effects might have contributed to the increase of TNF-α release and impaired liver regeneration. However, in vivo fluorescence microscopic analysis of regenerating livers revealed no significant decline of perfused sinusoids in EPO-treated animals in comparison to controls at all studied timepoints after hepatectomy (data not shown). Moreover, even the ten-fold lower dose of EPO with negligible hematopoiesis significantly diminished the regeneration capacity of the liver at the timepoint of maximal DNA synthesis, i.e. at 24 h after pHx. Thus the harmful effect of EPO in liver regeneration seems to be rather independent of its hematopoietic action.

In conclusion, enhanced release of TNF-α following multiple dosing of EPO after hepatectomy resulted in an increase of apoptotic cell death and a delay of liver regeneration. Careful consideration should therefore be made in repetitive pre- and post-operative rHuEPO administration in the setting of liver resection and transplantation.

## References

[1] Fausto N, Campbell JS, Riehle KJ (2006). Liver regeneration.. Hepatology.

[2] Fausto N, Riehle KJ (2005). Mechanisms of liver regeneration and their clinical implications.. J Hepatobiliary Pancreat Surg.

[3] Humar A, Kosari K, Sielaff TD, Glessing B, Gomes M (2004). Liver regeneration after adult living donor and deceased donor split-liver transplants.. Liver Transpl.

[4] Pomfret EA, Pomposelli JJ, Gordon FD, Erbay N, Lyn Price L (2003). Liver regeneration and surgical outcome in donors of right-lobe liver grafts.. Transplantation.

[5] Libbrecht L, Roskams T (2002). Hepatic progenitor cells in human liver diseases.. Semin Cell Dev Biol.

[6] Buemi M, Nostro L, Romeo A, Giacobbe MS, Aloisi C (2006). From the oxygen to the organ protection: erythropoietin as protagonist in internal medicine.. Cardiovasc Hematol Agents Med Chem.

[7] Sharples EJ, Thiemermann C, Yaqoob MM (2006). Novel applications of recombinant erythropoietin.. Curr Opin Pharmacol.

[8] Brines M, Cerami A (2006). Discovering erythropoietin's extra-hematopoietic functions: biology and clinical promise.. Kidney Int.

[9] Ghezzi P, Brines M (2004). Erythropoietin as an antiapoptotic, tissue-protective cytokine.. Cell Death Differ.

[10] Jelkmann W, Wagner K (2004). Beneficial and ominous aspects of the pleiotropic action of erythropoietin.. Ann Hematol.

[11] Jelkmann W (2004). Molecular biology of erythropoietin.. Intern Med.

[12] Vesey DA, Cheung C, Pat B, Endre Z, Gobe G (2004). Erythropoietin protects against ischaemic acute renal injury.. Nephrol Dial Transplant.

[13] Calvillo L, Latini R, Kajstura J, Leri A, Anversa P (2003). Recombinant human erythropoietin protects the myocardium from ischemia-reperfusion injury and promotes beneficial remodeling.. Proc Natl Acad Sci USA.

[14] Belayev L, Khoutorova L, Zhao W, Vigdorchik A, Belayev A (2005). Neuroprotective effect of darbepoetin alfa, a novel recombinant erythropoietic protein, in focal cerebral ischemia in rats.. Stroke.

[15] Johnson DW, Pat B, Vesey DA, Guan Z, Endre Z (2006). Delayed administration of darbepoetin or erythropoietin protects against ischemic acute renal injury and failure.. Kidney Int.

[16] Schmeding M, Boas-Knoop S, Lippert S, Ruehl M, Somasundaram R (2007). Erythropoietin promotes hepatic regeneration after extended liver resection in rats.. J Gastroenterol Hepatol.

[17] Seehofer D, Neumann UP, Schirmeier A, Carter J, Cho SY (2008). Synergistic effect of erythropoietin but not G-CSF in combination with curcumin on impaired liver regeneration in rats.. Langenbecks Arch Surg 2008.

[18] Higgins GM, Anderson RM (1931). Experimental pathology of the liver.. Arch Pathol.

[19] Cantré D, Schuett H, Hildebrandt A, Dold S, Menger MD (2008). Nitric oxide reduces organ injury and enhances regeneration of reduced-size livers by increasing hepatic arterial flow.. Br J Surg.

[20] Kountouras J, Boura P, Lygidakis NJ (2001). Liver regeneration after hepatectomy.. Hepatogastroenterology.

[21] Nadalin S, Testa G, Malago M, Beste M, Frilling A (2004). Volumetric and functional recovery of the liver after right hepatectomy for living donation.. Liver Transpl.

[22] Bardet V, Junior AP, Coste J, Lecoq-Lafon C, Chouzenoux S (2006). Impaired erythropoietin production in liver transplant recipients: the role of calcineurin inhibitors.. Liver Transp.

[23] Katavetin P, Tungsanga K, Eiam-Ong S, Nangaku M (2007). Antioxidative effects of erythropoietin.. Kidney Int.

[24] Ehrenreich H, Hasselblatt M, Dembowski C, Cepek L, Lewczuk P (2002). Erythropoietin therapy for acute stroke is both safe and beneficial.. Mol Med.

[25] Macdougall IC, Roberts DE, Coles GA, Williams JD (1991). Clinical pharmacokinetics of epoetin (recombinant human erythropoietin).. Clin Pharmacokinet.

[26] Spivak JL, Hogans BB (1989). The in vivo metabolism of recombinant human erythropoietin in the rat.. Blood.

[27] Vairano M, Dello Russo C, Pozzoli G, Battaglia A, Scambia G (2002). Erythropoietin exerts anti-apoptotic effects on rat microglial cells in vitro.. Eur J Neurosci.

[28] Okada T, Sawada T, Kubota K (2007). Asialoerythropoietin has strong renoprotective effects against ischemia-reperfusion injury in a murine model.. Transplantation.

[29] Zhang F, Wang S, Cao G, Gao Y, Chen J (2007). Signal transducers and activators of transcription 5 contributes to erythropoietin-mediated neuroprotection against hippocampal neuronal death after transient global cerebral ischemia.. Neurobiol Dis.

[30] Zhande R, Karsan A (2007). Erythropoietin promotes survival of primary human endothelial cells through PI3K-dependent, NF-kappaB-independent upregulation of Bcl-xL.. Am J Physiol Heart Circ Physiol.

[31] Le Minh K, Klemm K, Abshagen K, Eipel C, Menger MD (2007). Attenuation of inflammation and apoptosis by pre- and posttreatment of darbepoetin-alpha in acute liver failure of mice.. Am J Pathol.

[32] Yilmaz S, Ates E, Tokyol C, Pehlivan T, Erkasap S (2004). The protective effect of erythropoietin on ischaemia/reperfusion injury of liver.. HPB (Oxford).

[33] Ogata T, Yamashita K, Horiuchi H, Okuda K, Todo S (2008). A novel tumor necrosis factor-alpha suppressant, ONO-SM362, prevents liver failure and promotes liver regeneration after extensive hepatectomy.. Surgery.

[34] Diehl AM, Rai R (1996). Review: regulation of liver regeneration by pro-inflammatory cytokines.. J Gastroenterol Hepatol.

[35] Nagy LE (2004). Stabilization of tumor necrosis factor-alpha mRNA in macrophages in response to chronic ethanol exposure.. Alcohol.

[36] Kawashima Y, Takeyoshi I, Otani Y, Koibuchi Y, Yoshinari D (2001). FR167653 attenuates ischemia and reperfusion injury of the rat lung with suppressing p38 mitogen-activated protein kinase.. J Heart Lung Transplant.

[37] Nagata Y, Moriguchi T, Nishida E, Todokoro K (1997). Activation of p38 MAP kinase pathway by erythropoietin and interleukin-3.. Blood.

[38] Rafiee P, Shi Y, Su J, Pritchard KA, Tweddell JS (2005). Erythropoietin protects the infant heart against ischemia-reperfusion injury by triggering multiple signaling pathways.. Basic Res Cardiol.

[39] Chen GG, Lai PB, Hu X, Lam IK, Chak EC (2002). Negative correlation between the ratio of Bax to Bcl-2 and the size of tumor treated by culture supernatants from Kupffer cells.. Clin Exp Metastasis.

[40] Michalopoulos GK, DeFrances MC (1997). Liver regeneration Science..

[41] Tsutsumi R, Kamohara Y, Eguchi S, Azuma T, Fujioka H (2004). Selective suppression of initial cytokine response facilitates liver regeneration after extensive hepatectomy in rats.. Hepatogastroenterology.

[42] Mohammed FF, Smookler DS, Taylor SE, Fingleton B, Kassiri Z (2004). Abnormal TNF activity in Timp3-/- mice leads to chronic hepatic inflammation and failure of liver regeneration.. Nat Genet.

[43] Wiessner C, Allegrini PR, Ekatodramis D, Jewell UR, Stallmach T (2001). Increased cerebral infarct volumes in polyglobulic mice overexpressing erythropoietin.. J Cereb Blood Flow Metab.

